# Supplementary material for: Decrease in the prevalence of hepatitis B and D virus infections in an endemic area in Peru 23 years after the introduction of the first pilot vaccination program against hepatitis B
Source: PLoS One. 2020 Aug 6;15(8):e0236993. doi: 10.1371/journal.pone.0236993 (PMC7410281; doi:10.1371/journal.pone.0236993)
Supplement: Attachment — Submitted filename: Review PLos jan 2020.docx [file pone.0236993.s002.docx]

The study performed by Cabezas and colleagues demonstrated HBV prevalence reduction 23 years after the introduction of a pilot vaccination program in the high prevalence area of Abancay, Peru. The study has merit, however, some points should be addressed before publication.

Keywords (line 40): It would be more informative to add Peru as a keyword instead Abancay.

Material and Methods:

The number and exact location of study population is not clear. How many people from each district/conglomerate were selected? It is partially set in table 1, but should be better explained. How many individuals are from urban areas and how many are from rural areas? Please, clarify these points. I suggest this information to be added to Figure 1 to make it more informative. Figure 1 could contain more details about the study population (number of subjects/district and district locations). As it stands, it is very similar to other figures available on various websites (wikipedia etc).

Line 91: What the authors mean as “each geographical area”? It should be better explained. I suggest it to be placed in figure 1.

Line 92: The authors mean “A total of 113 conglomerates comprising 32 inhabitants **each** ? If yes, please add this word.

Lines 95-95: In the sentence *“In cases of rejections or losses, the houses were* ***not*** *replaced by the next eligible house on the list”*, is the word ‘not’ placed correctly? If yes, the sentence is not relevant. If no, please make the correction.

Results:

Line 133: How many individuals were recruited and what was the loss? Please, provide this information.

Figure 2: Please, rephrase the caption. It is not clear, the text could be improved. Also, on the x-axis of the chart, the term “*Carrier rate of HBsAg* (%)” is confusing. Please replace to "HBsAg carriers (%)" or just "HBsAg (%)".

Lines 135-137: The sentence “*A decreased trend in the carrier rates of HBsAg, compared with those reported in previous studies, in populations of Abancay 24 years since the introduction of the first pilot vaccination program against HBV was observed (9.8% in 1991 vs. 1.2% in 2014 (Fig 2) [11]*.” is more a discussion than a result. Also, in other points of the paper, the authors set 23 instead 24 years since the first vaccination program. Please, standardize the time interval.

Line 143: How was the viral load and serological profile of the HBV acute infected subject?

Lines 146-147: Did the anti-HDV carriers present higher HBV viral loads?

Lines 148-154: I suggest to replace *“carrier rates of HBsAg, anti-HBc and anti-HBs”* by “HBsAg anti-HBc and anti-HBs prevalences” or “HBsAg anti-HBc and anti-HBs positivity”.

Discussion:

Lines 169-170: *“In this study, we also observed a decline in the prevalence of HDV among HBsAg carriers, from 9% in 1990 [11] to 5.2% in 2014”*. Was this decrease statistically significative?

Lines 186-190: This is probably related to the fact that these individuals either did not have access to vaccination or did not respond to the vaccine.One possible explanation is that those with repeated HBV exposure no longer became infected and hence did not mount protective levels of anti-HBs, possibly due to either a defense mechanism or changes in their immune response”.If I understood correctly, in this sentence the authors attributed the anti-HBs levels (that might be due to low vaccination response) to repeated HBV exposures over time. It is a quite speculative and unlikely to happen, once individuals exposed to HBV usually have positivity for both anti-HBs and anti-HBc serological markers. Please clarify the sentence.

Other comments:

-Is there any difference in HBV prevalences between urban and rural areas?

-Based on table one, individuals from nine districts have been recruited, most of them to Abancay, where the highest prevalences of HBV serological markers were observed. It can lead to a bias and should be better discussed.

-The age groups showed in table 1 could be standardized and better explored/discussed

-The authors performed molecular tests (HBV viral loads) but did not provide any discussion exploring these points.
