# Supplementary material for: Decrease in the prevalence of hepatitis B and D virus infections in an endemic area in Peru 23 years after the introduction of the first pilot vaccination program against hepatitis B
Source: PLoS One. 2020 Aug 6;15(8):e0236993. doi: 10.1371/journal.pone.0236993 (PMC7410281; doi:10.1371/journal.pone.0236993)
Supplement: Attachment — Submitted filename: Response-to-review_PONE-D-19-35864R1.docx [file pone.0236993.s004.docx]

**Response-to-reviewers: Manuscript PONE-D-19-35864R1**

We thank the Reviewers for their comments and constructive criticism, we believe that the quality of our manuscript has been significantly improved. We have revised our paper in a point-by-point manner. Modifications are in yellow text.

Reviewer #2: Thanks for the authors responses to my comments. As responded by the author, the novity of this study was the results about anti-HBc (~42%) and anti-HBs (~39%). Based on the low prevalence of HBsAg, I assume the increasing prevalence of anti-HBc with age suggested natural immunity while the decreasing prevalence of anti-HBs with age correlated with the immunization program. But such kind of summary was not found in the current paper. I agree the reduction of HBsAg was consistent with previous study which showed validity of the data. But I would recommend the author wrote more about the novel results and the interpretation of them.

Response: Thank you for this suggestion; We have corrected these paragraphs in Discussion section (lines 205-214 and lines 218-228).

My other concern is still about the statistical test and presentation of the results. For instance,
1. Table 1. As the study showed the prevalence, there should not be any 95% CI include negative values. But this is the case in a lot of numbers.

Response: Thank you for this suggestion; we have corrected Table 1.

2. Table 2. The author used chi-square test, but what the reference group and how large are the samples for each group. As this is not an adjusted analysis, why did not the authors combine Table 2 with Table 1? By doing this, more statistical comparison could also be provided.

Response: Thank you for this suggestion; we have combine Table 2 with Table 1 “Prevalence rates of HBsAg and anti-HBc”, include the p-values. For a better understanding of the readers, we have also include a Table 2 “prevalence rates of anti-HBs at protective levels (i.e., ≥10 mIU/ml) in individuals who were HBsAg and anti-HBc negative”. We have also re-estimated the prevalence rates of anti-HBs (lines 160-167 and Table 2).
